# Ácido Esteárico (mas não o Ácido Palmítico) está Associado a Biomarcadores Inflamatórios e de Disfunção Endotelial em Indivíduos em Risco Cardiovascular

**DOI:** 10.36660/abc.20220598

**Published:** 2023-08-10

**Authors:** Gustavo Henrique Ferreira Gonçalinho, Geni Rodrigues Sampaio, Rosana Aparecida Manólio Soares-Freitas, Nágila Raquel Teixeira Damasceno

**Affiliations:** 1 Universidade de São Paulo Departamento de Nutrição São Paulo SP Brasil Universidade de São Paulo – Departamento de Nutrição , São Paulo , SP – Brasil

**Keywords:** Ácidos Graxos, Endotélio, Inflamação, Biomarcadores, Doenças Cardiovasculares

## Abstract

**Fundamento:**

Vários estudos têm associado o consumo de ácidos graxos saturados (AGSs) com risco cardiovascular, mas ainda existem muitas controvérsias. A maioria desses estudos avaliou os efeitos do ácido palmítico sobre lipídios circulantes. O ácido esteárico geralmente apresenta um efeito neutro sobre os lipídios sanguíneos, mas faltam estudos clínicos avaliando sua relação com marcadores de inflamação e de disfunção endotelial.

**Objetivos:**

Avaliar a associação de AGSs das hemácias (ácido palmítico e ácido esteárico) com biomarcadores inflamatórios e de disfunção endotelial circulantes.

**Métodos:**

Estudo transversal que incluiu 79 adultos de ambos os sexos com pelo menos um fator de risco cardiovascular, mas sem eventos prévios (infarto agudo do miocárdio ou acidente vascular cerebral). Biomarcadores plasmáticos – lipídios, marcadores glicometabólicos, proteína C ultrassensível (PCR-us), Interleucina 6 (IL-6), Interleucina 10 (IL-10), Fator de Necrose Tumoral-α (TNF-α), Proteína quimioatraente de Monócitos 1 (MCP-1) – e ácidos graxos das hemácias (ácidos palmítico e esteárico) foram analisados. As associações foram avaliadas por análises de correlações e regressões lineares múltiplas, com significância estatística estabelecida em p<0,05.

**Resultados:**

O ácido palmítico não apresentou associações com fatores de risco cardiovasculares ou com marcadores inflamatórios. Por outro lado, o ácido esteárico foi inversamente correlacionado com PCR-us, IL-6 e TNF-α, mas independentemente associado com PCR-us, IL-6, e TNF-α.

**Conclusão:**

O ácido esteárico está associado com biomarcadores inflamatórios e disfunção endotelial em indivíduos com um ou mais fatores de risco cardiovascular.

## Introdução

O papel da inflamação nas doenças cardiovasculares (DCVs) tem ganhado bastante ênfase na literatura. Pacientes em terapia hipolipemiante intensiva com estatinas, ezetimiba, e inibidores de PCSK9 podem apresentar o chamado “risco inflamatório residual”, no qual pacientes com níveis mais altos de marcadores inflamatórios, tais como fator de necrose tumoral-α (TNF-α), interleucina-6 (IL-6) e proteína C reativa (PCR), mesmo com redução dos níveis de colesterol, podem apresentar mais eventos cardiovasculares em comparação a pacientes com níveis mais baixos. ^1^ Vários biomarcadores de inflamação foram associados com a incidência, a prevalência, a gravidade, e o prognóstico de DCVs. Eles podem refletir a DCV de uma perspectiva diferente daquela de fatores de risco tradicionais, uma vez que muitos estudos mostraram independência na associação desses marcadores. ^2^ Ainda, o aumento desses marcadores inflamatórios circulantes foi associado com níveis elevados de molécula de adesão celular vascular-1 (VCAM-1), molécula de adesão intercelular-1 (ICAM-1), e E-selectina, indicadores de disfunção endotelial. Tal fato pode explicar a relação entre inflamação e DCV; ^3 , 4^ assim, marcadores inflamatórios como PCR e IL-6 também podem indicar disfunção endotelial. ^2 - 4^

A evidência atual sugere que a ingestão elevada de ácidos graxos saturados (AGSs) está associada a um risco cardiovascular aumentado, ^5^ e limitar o consumo de AGSs para redução desse risco é ainda recomendado pelas diretrizes de nutrição da American Heart Association ^6^ e da Sociedade Brasileira de Cardiologia. ^7^ Os mecanismos pelos quais os AGSs contribuem para a progressão da aterosclerose incluem principalmente o aumento nos níveis sanguíneos de colesterol total, lipoproteína de baixa densidade (LDL), e triglicerídeos, ^8^ e a inflamação. ^9 - 11^

Entre os mecanismos relatados na literatura, mostrou-se que os AGSs são ativadores de receptores do tipo Toll (TLRs) em macrófagos, ativando a via de sinalização inflamatória e, subsequentemente, disfunções imunometabólicas encontradas nas doenças cardiometabólicas. ^12^ Evidência clínica de como os AGSs influenciam a inflamação e consequentemente o risco cardiovascular permanece controverso devido aos diferentes métodos aplicados, ^12^ e faltam estudos sobre a influência desse AGS sobre biomarcadores da função endotelial e inflamação. ^12^ Inquéritos alimentares, comumente usados em estudos que avaliaram os efeitos dos AGSs sobre a saúde, contêm vários vieses, e os ácidos graxos das hemácias têm sido usados como biomarcadores do estado nutricional desses nutrientes, ^13^ podendo fornecer evidências mais consistentes sobre como os AGSs estão associados com inflamação e função endotelial em humanos.

Assim, o objetivo do presente estudo é avaliar a associação dos AGSs das hemácias (ácidos palmítico e esteárico) com biomarcadores circulantes da inflamação e disfunção endotelial em indivíduos com fatores de risco cardiovasculares, mas sem DCV estabelecida.

## Métodos

### Delineamento e participantes do estudo

**Figura 1:**
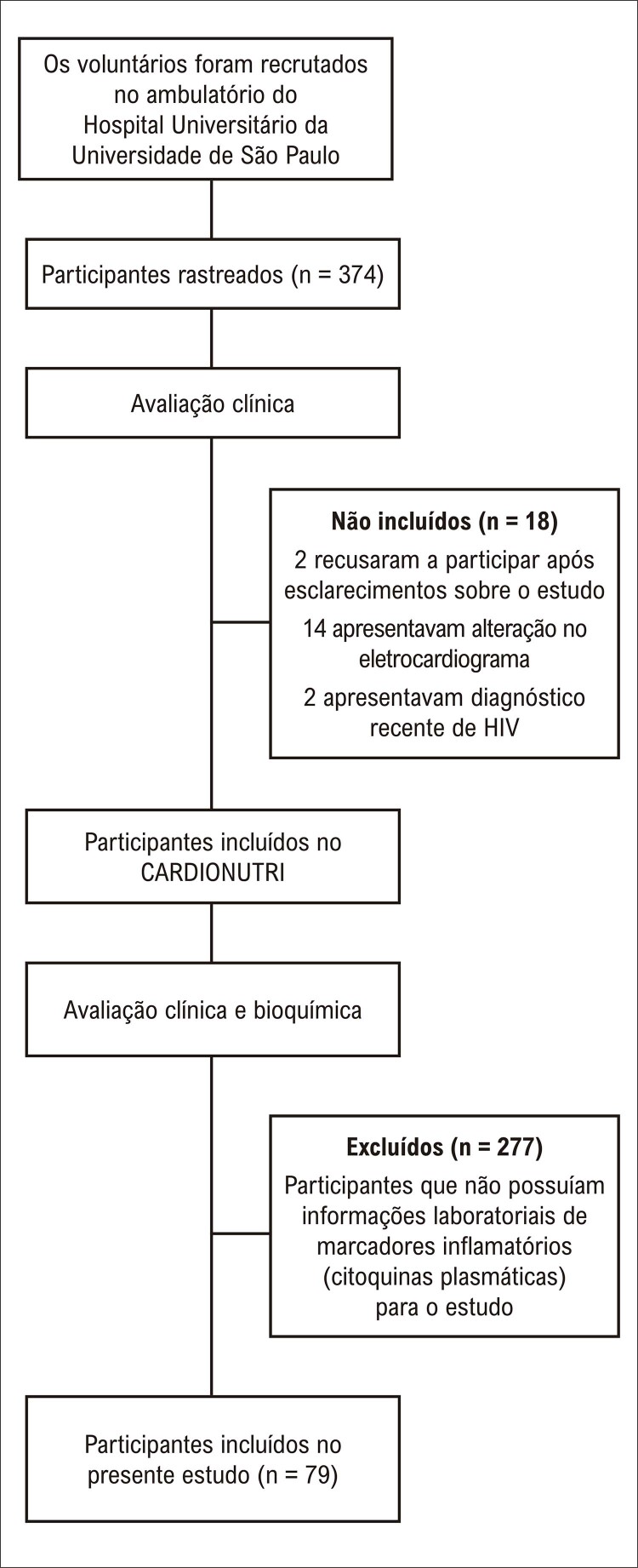
– Gráfico do estudo.

Este foi um estudo transversal dos dados basais do ensaio clínico CARDIONUTRI ((ReBEC: RBR-2vfhfv). Os participantes foram recrutados do ambulatório do hospital da Universidade de São Paulo. Os critérios de inclusão foram indivíduos de ambos os sexos, idade entre 30 e 74 anos, com ao menos um fator de risco cardiovascular, e sem eventos cardiovasculares prévios (infarto agudo do miocárdio ou acidente vascular cerebral). Os critérios de exclusão foram indivíduos com doenças agudas ou doenças crônicas graves, doenças infecciosas, mulheres grávidas e/ou lactantes. Os indivíduos rastreados passaram por uma breve entrevista por telefone para avaliação dos critérios de inclusão e exclusão. Ainda, os indivíduos foram submetidos a uma eletrocardiografia conduzida por um cardiologista, e aqueles com alterações sugestivas de eventos cardiovasculares prévios foram excluídos. Um total de 374 indivíduos foram recrutados para o estudo em 2011 e 2012. Dois indivíduos recusaram a participar após serem informados sobre o delineamento do estudo. Quatorze pacientes foram excluídos por apresentarem alterações no eletrocardiograma e dois por diagnóstico recente de infecção pelo HIV. Ao final do recrutamento, 356 indivíduos foram incluídos no ensaio CARDIONUTRI. Para a análise do presente estudo, foram incluídos somente pacientes com informações laboratoriais sobre marcadores inflamatórios (citoquinas plasmáticas), resultando em 79 indivíduos ( Figura 1 ).

### Avaliação clínica e nutricional

A história clínica de doenças crônicas não transmissíveis e uso atual de medicamentos foram relatadas pelos pacientes. O exame físico incluiu medida do Índice de Massa Corporal (IMC), circunferência da cintura, e pressão arterial. A ingestão dietética foi obtida por três recordatórios alimentares de 24 horas e avaliada no programa Food Processor (ESHA Research, 2012), com subsequente ajuste quanto à energia. ^14^ O risco cardiovascular foi avaliado pelo escore de risco Framingham, ^15 , 16^ e os indivíduos foram classificados com risco baixo, moderado ou alto. Diabetes foi considerado um equivalente da Doença Arterial Coronariana (DAC). ^17^

### Análise bioquímica

Amostras de sangue foram coletadas após jejum de 12 horas em tubos contendo EDTA (1.0 mg/mL), e os eritrócitos foram separados do plasma por centrifugação, e ambos congelados a -80 ^o^ C, imediatamente após a coleta. Inibidores de protease (10 µg/mL de aprotinina, 10 µg/mL de benzamidina, e 5 µg/mL de fenilmetilsulfonil) e hidroxitolueno butilado (BHT, 100 µg/mL) foram adicionados para preservar as amostras. Todas as amostras foram divididas em alíquotas para evitar ciclos repetidos de degelo e armazenados a -80 ^o^ C até as análises. Concentrações plasmáticas de colesterol total, lipoproteína de alta densidade (HDL), triglicerídeos, glicose, (Labtest Diagnostica SA, MG, Brasil), ácidos graxos não esterificados (AGNEs), apolipoproteínas (Apo) A-I e B (Wako Chemicals USA Inc., Richmond, VA, USA), e proteína C reativa ultrassensível (PCR-us) foram medidas usando kits comerciais. Valores plasmáticos de Interleucina (IL)-6, IL-10, proteína quimioatraente de monócitos 1 (MCP-1) e Fator de Necrose Tumoral-α (TNF-α) foram medidos usando um kit de ensaio de imunoabsorção enzimática (ELISA) Bio-PlexTM Human Cytokine 17-plex (Bio-Rad Laboratories, CA, EUA). A insulina sérica foi medida pelo kit de ELISA para insulina humana (Life Technologies, NY, EUA). O LDL foi calculado usando a equação de Friedewald. ^18^ A sensibilidade à insulina foi medida pelo modelo de avaliação da homeostase da resistência à insulina (HOMA-IR), e resistência insulínica foi diagnosticada foi diagnosticada se os indivíduos apresentassem qualquer das seguintes condições: IMC > 28,9 Kg/m ^2^ , HOMA-IR >4,65, ou IMC >27,5 kg/m ^2^ e HOMA-IR >3,60. ^19^

### Análise dos ácidos graxos das hemácias

A análise dos ácidos graxos de hemácias foi realizada por cromatografia gasosa conforme descrito em outro estudo. ^13^ Após a separação do plasma, 300mL de hemácias foram lavadas quatro vezes com 5 mL de solução salina tamponada com fosfato (PBS, pH 7,4). O precipitado foi transferido para tubos com tampas de rosca, aos quais foram adicionados 1,75 mL de metanol, 50 µL de solução padrão interna contendo ácido tetradecanóico (C13:0)/1 mL hexano, e 100 µL de cloreto de acetila. A solução foi agitada em um vórtex e aquecida em banho maria a 90 ^o^ C por uma hora. Em seguida, 1,5 mL de hexano foi adicionado, e a solução homogeneizada por um minuto. As amostras foram centrifugadas a 1500 × g, a 4 ^o^ C por dois minutos, e 800 µL do sobrenadante foi transferido para outro tubo. Essa etapa foi repetida com a adição de 750 µL de hexano. Os tubos contendo os sobrenadantes coletados foram colocados em um concentrador centrífugo a 40 ^o^ C por 20 minutos. Em seguida, ésteres metílicos de ácidos graxo foram dissolvidos em 150 µL de hexano e transferidos a um *insert* de vidro encaixado em um *vial* . No total, 19 ácidos graxos de hemácias foram descritos, os quais incluíram AGSs, ácidos graxos monoinsaturados (AGMI), e ácidos graxos poli-insaturados (AGPI). Entre os ácidos graxos de hemácias, analisamos os ácidos palmítico, esteárico, eicosanoico, docosanoico, lignocérico, palmitoleico, oleico, gondoico, erúcico, nervônico, linoleico, γ-linoleico, eicosadienoico, dihomo-γ-linolênico, araquidônico, 13,16-docosadienoico, α-linoleico, eicosapentaenoico, e ácido docosahexaenoico. No presente estudo, focamos nossas análises nos AGSs, mais especificamente nos ácidos esteárico e palmítico, os quais foram os AGSs mas predominantes nos eritrócitos.

### Análise estatística

A distribuição das variáveis foi avaliada pelo teste de Kolmogorov-Smirnov. As características das amostras são apresentadas em média e desvio padrão (DP) ou mediana e intervalo interquartil (IIQ) para as variáveis quantitativas, dependendo da distribuição das variáveis, e como frequência (n) e porcentagem (%) para as variáveis categóricas.

A relação dos AGSs com os parâmetros cardiometabólicos e citocinas foi avaliada por correlações de Pearson e de Spearman, dependendo da distribuição das variáveis.

Para avaliar a influência dos ácidos palmítico e esteárico sobre biomarcadores de inflamação, foram aplicadas múltiplas regressões lineares usando os biomarcadores inflamatórios (PCR-us, IL-6, IL-10, MCP-1, TNF-α) como variáveis dependentes, e o ácido palmítico ou esteárico como a variável independente além das variáveis de ajuste (idade, sexo, tabagismo, colesterol total, pressão arterial sistólica, glicemia e IMC). Todos os pressupostos da regressão foram preenchidos (isto é, ausência de multicolinearidade, homoscedasticidade, erros independentes e com distribuição normal, independência das variáveis de desfechos, e linearidade das variáveis).

Todos os testes foram bicaudais, com p<0,05 considerado como estatisticamente significativo, e conduzidos no programa SPSS versão 20.0.

## Resultados

As características dos participantes estão resumidas nas Tabelas 1 e S1. A amostra foi composta predominantemente por mulheres (59.5%), e a idade média foi 51,0 ± 10,3 anos. A prevalência de doenças crônicas autorrelatadas na amostra foi de 60,8 % de indivíduos com hipertensão, 52,2% de dislipidemia, e 25,3% de diabetes mellitus tipo 2.

Quanto ao escore de risco Framingham, a maioria dos participantes foi classificada como em alto risco cardiovascular (51,9%), seguido de risco moderado (34,2%) e baixo risco (13,9%). A amostra apresentou vários fatores de risco cardiovasculares, tais como valores elevados de IMC, circunferência da cintura, colesterolemia, glicemia, insulinemia, PCR-us e HOMA-IR, e baixo HDL ( Tabela 1 ).

**Tabela 1:** – Características clínicas e bioquímicas dos participantes do estudo

Variáveis	Média/Mediana	DP/IIQ
Idade, anos	51,0	10,3
Índice de massa corporal, Kg/m ^2^	31,5	6,1
Circunferência da cintura, cm	101,5	13,1
Pressão arterial sistólica, mmHg	135,2	19,2
Pressão arterial diastólica, mmHg	81,8	9,9
Frequência cardíaca, bpm	67,8	13,0
Colesterol total, mg/dL	199,8	43,9
HDL-c, mg/dL	35,8	10,2
LDL-c, mg/dL	134,8	41,4
Colesterol total:HDL-c	6,0	4,0 – 7,0
Colesterol não-HDL, mg/dL	164,4	45,0
ApoA-I, mg/dL	126,9	25,7
ApoB, mg/dL	101,8	26,9
Triglicerídeos, mg/dL	129,0	94,0 – 188,0
Ácidos graxos não esterificados, mEq/dL	0,62	0,25
Glicose, mg/dL	96,0	89,0 – 108,0
Insulina, μUI/mL	17,7	8,4
HOMA-IR	4,4	2,4
PCR-us, mg/L	2,8	1,2 – 5,6
IL-6, pg/mL	5,3	3,3 – 9,0
IL-10, pg/mL	4,0	2,8 – 10,5
MCP-1, pg/mL	15,1	10,5 – 21,6
TNF-α, pg/mL	15,2	8,2 – 25,9

HDL: lipoproteína de alta densidade; LDL: lipoproteína de baixa densidade; Apo: apolipoproteína; HOMA-IR: modelo de avaliação da homeostase da resistência à insulina; IL: interleucina; PCR-us: proteína C Reativa Ultrassensível; TNF-α: fator de necrose tumoral-α, MCP-1: proteína quimioatraente de Monócitos 1; DP: desvio padrão; IIQ: intervalo interquartil.

O perfil dos ácidos graxos das hemácias está descrito na Tabela 2 . Os ácidos graxos mais abundantes foram os ácidos palmítico e esteárico. O AGMI mais abundante foi o ácido oleico (C18:1 n-9). Entre os AGPI, os mais frequentes foram o ácido linoleico e o ácido araquidônico da família n-6 e o ácido docosahexaenóico da família n-3.

**Tabela 2:** – Perfil dos ácidos graxos das hemácias dos participantes do estudo

Variáveis	Média/Mediana	DP/IIQ
**AGS, %**		
C16:0 (ácido palmítico)	44,02	4,87
C18:0 (ácido esteárico)	26,12	4,45
C20:0 (ácido eicosanóico)	0,73	0,18
C22:0 (ácido docosanoico)	1,29	0,51
C24:0 (ácido lignocérico)	0,27	0,14 – 0,77
**AGMI, %**		
C16:1 n-7 (ácido palmitoleico)	0,33	0,22 – 0,76
C18:1 n-9 (ácido oleico acid)	8,99	3,71
C20:1 n-9 (ácido gondoico)	0,08	0,05 – 0,14
C22:1 n-9 (ácido erúcico)	0,14	0,10 – 0,21
C24:1 n-9 (ácido nervônico)	1,36	0,67
**AGPI n-6, %**		
C18:2 n-6 (ácido linoleico)	4,34	1,91
C18:3 n-6 (ácido γ-linoleico)	0,17	0,12 – 0,24
C20:2 n-6 (ácido eicosadienoico)	0,10	0,07 – 0,17
C20:3 n-6 (ácido dihomo-γ-linolênico)	0,54	0,36
C20:4 n-6 (ácido araquidônico)	3,43	2,60
C22:2 n-6 (ácido 13,16-docosadienoico)	0,39	0,21
n-6 total	9,18	4,07
**AGPI n-3, %**		
C18:3 n-3 (ácido α-linolenico)	1,64	1,01 – 2,28
C20:5 n-3 (ácido eicosapentaenoico)	0,24	0,14
C22:6 n-3 (ácido docosahexaenoico)	3,91	1,31
n-3 total	6,16	2,03

AGMI: ácidos graxos monoinsaturados; AGPI: ácidos graxos poli-insaturados; DP: desvio padrão; IIQ: intervalo interquartil.

A composição nutricional da dieta dos participantes e a sua correlação com os marcadores inflamatórios estão descritas nas Tabelas S2 e S3. O consumo médio de AGSs foi 10,2±3,9%, sendo o consumo de ácido palmítico de 6,4 ± 4,0g e o de ácido esteárico 3,0 ± 2,4 g. Não houve correlação estatisticamente significativa entre AGS dietético e marcadores inflamatórios.

As correlações entre AGS das hemácias (ácidos palmítico e esteárico) estão descritas na Tabela 3 . Colesterol total, colesterol não-HDL, razão colesterol total: colesterol HDL, apoB, e triglicerídeos correlacionaram-se inversamente com o ácido esteárico. No entanto, PCR-us, IL-6, IL-10, MCP-1 e TNF-α correlacionaram-se positivamente com o ácido esteárico.

**Tabela 3:** – Correlações entre ácidos graxos das hemácias e biomarcadores cardiovasculares

Variáveis	Ácido palmítico		Ácido esteárico	
	r	Valor p	r	Valor p
Sexo	0,079	0,490	0,197	0,082
Idade, anos	0,062	0,585	0,163	0,151
Índice de massa corporal, Kg/m ^2^	0,013	0,907	-0,036	0,756
Circunferência da cintura, cm	0,021	0,857	-0,076	0,506
Pressão arterial sistólica, mmHg	0,034	0,767	-0,113	0,323
Pressão arterial diastólica, mmHg	0,024	0,833	-0,151	0,184
Frequência cardíaca, bpm	0,049	0,667	0,072	0,531
Colesterol total, mg/dL*	-0,032	0,779	-0,253	0,024
HDL-c, mg/dL	0,089	0,434	0,198	0,080
LDL-c, mg/dL	-0,088	0,453	-0,211	0,069
Colesterol total:HDL-c	-0,083	0,468	-0,330	0,003
Colesterol não-HDL, mg/dL	-0,052	0,649	-0,292	0,009
ApoA-I, mg/dL	0,155	0,173	-0,023	0,843
ApoB, mg/dL	0,009	0,936	-0,290	0,009
Triglicerídeos, mg/dL*	-0,011	0,927	-0,244	0,030
Ácidos graxos não esterificados, mEq/dL	0,283	0,016	-0,007	0,954
Glicose, mg/dL*	-0,114	0,317	-0,034	0,768
Insulina, μUI/mL	0,133	0,298	-0,220	0,083
HOMA-IR	0,093	0,467	-0,210	0,099
PCR-us, mg/L*	0,114	0,325	0,286	0,012
IL-6, pg/mL*	0,017	0,881	0,340	0,002
IL-10, pg/mL*	0,029	0,833	0,357	0,007
MCP-1, pg/mL*	0,034	0,769	0,245	0,030
TNF-α, pg/mL*	0,009	0,938	0,364	0,002

A análise de correlação foi realizada usando o teste de Pearson para variáveis paramétricas e o teste de Spearman (*) para variáveis não paramétricas. HDL: lipoproteína de alta densidade; LDL: lipoproteína de baixa densidade; Apo: apolipoproteína; HOMA-IR: modelo de avaliação da homeostase da resistência à insulina; IL: interleucina; PCR-us: proteína C reativa ultrassensível; TNF-α: fator de necrose tumoral-α; MCP-1: proteína quimioatraente de Monócitos 1; DP: desvio padrão; IIQ: intervalo interquartil.

Nos modelos de regressão linear múltipla ( Tabela 4 ), o ácido palmítico não apresentou associação estatisticamente significativa com nenhum dos biomarcadores inflamatórios, ao passo que o ácido esteárico foi um fator independentemente associado PCR-us, IL-6, e TNF-α quando ajustado por idade, sexo, tabagismo, colesterol total, pressão arterial sistólica, glicemia e IMC.

**Tabela 4:** – Associações entre ácidos graxos das hemácias e biomarcadores de inflamação e de disfunção endotelial circulantes

Variáveis dependentes	R2	β	SE	IC95% para β		Valor p
				Inferior	Superior	
**Ácido palmítico (C16:0)**						
PCR-us	0,079	0,165	0,162	-0,158	0,489	0,311
IL-6	0,110	0,132	1,789	-3,438	3,702	0,941
IL-10	0,127	0,728	2,134	-3,589	5,024	0,735
MCP-1	0,091	0,399	2,184	-3,957	4,756	0,855
TNF-α	0,127	0,112	1,279	-2,445	2,669	0,930
**Ácido esteárico (C18:0)**						
PCR-us	0,162	0,545	0,194	0,157	0,933	**0,007**
IL-6	0,173	4,620	2,026	0,577	8,664	**0,026**
IL-10	0,186	4,370	2,359	-0,379	9,120	0,070
MCP-1	0,116	3,584	2,526	-1,454	8,623	0,160
TNF-α	0,194	3,230	1,443	0,345	6,116	0,029

Modelos de regressão linear múltipla ajustados por idade, sexo, tabagismo, colesterol total, pressão arterial sistólica, pressão arterial, glicemia, e índice de massa corporal. Os modelos foram compostos por uma das variáveis dependentes – Proteína C Reativa Ultrassensível (PCR-us), Interleucina-6 (IL-6), Interleucina-10 (IL-10), Proteína quimioatraente de Monócitos 1 (MCP-1) e Fator de Necrose Tumoral-α (TNF-α) e variáveis independentes (ácido palmítico ou ácido esteárico e variáveis de ajuste). IC95%: intervalo de confiança de 95%.

## Discussão

O principal achado de nosso estudo ( Figura Central ) foi que a associação independente entre ácido esteárico e biomarcadores de disfunção endotelial e inflamação, apesar das correlações inversas com lipídios aterogênicos em indivíduos com fatores de risco cardiovascular sem DCV. Ainda, o ácido palmítico não mostrou associação significativa com nenhum dos biomarcadores avaliados.

**Figura Central:**
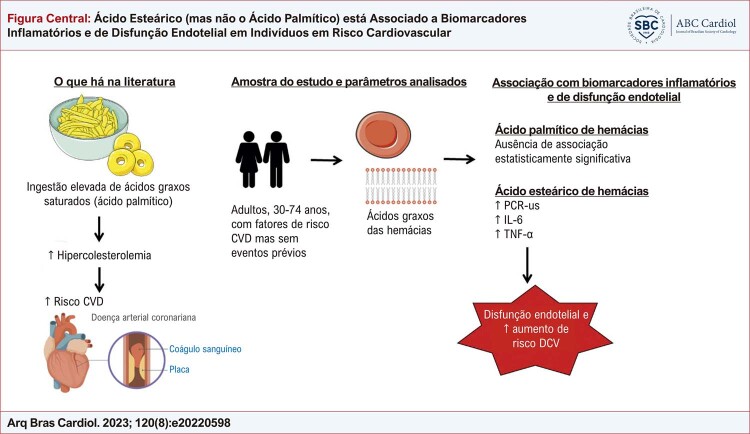
: Ácido Esteárico (mas não o Ácido Palmítico) está Associado a Biomarcadores Inflamatórios e de Disfunção Endotelial em Indivíduos em Risco Cardiovascular

DCV: doença cardiovascular; PCR-us: proteína C ultrassensível; IL-6: Interleucina 6.

Limitar a ingestão de AGS para diminuir o risco cardiovascular é uma recomendação das diretrizes nutricionais recentemente publicadas. ^6 , 7^ Uma meta-análise de Ensaios Clínicos Randomizados (ECRs) mostrou que a ingestão de óleo de palma rico em ácido palmítico aumentou os níveis de LDL em 9mg/dL em comparação a óleos vegetais pobres em AGS. ^20^ Tal fato pode ser traduzido em um risco 6% maior de Doença Arterial Coronariana (DAC). ^21^ Outra meta-análise mostrou que uma redução no consumo de AGSs diminuiu o nível de LDL em 5mg/dL em crianças e adolescentes. ^22^ O mais importante, esses efeitos ocorrem quando a faixa de ingestão dos ácidos graxos encontra-se entre 20 e 30% do total de energia ingerida. Quando a ingestão de ácidos graxos é maior que 30%, a magnitude do efeito do AGS de elevar os níveis de LDL aumentou para 24 mg/dL. ^20^ Contudo, outros estudos mostraram fracas associações entre a ingestão de AGS e desfechos cardiovasculares. ^23 , 24^ Tais controvérsias poderiam ser explicadas pela substituição dos AGSs por carboidratos refinados, ^25^ e a comparação dos efeitos dos AGSs com os de ácidos graxos trans, gordura animal, ou óleo de coco, ^20^ dificultaria a avaliação do real efeito dos AGSs. Ainda, a maioria dos estudos observacionais não distinguem os efeitos do ácido palmítico e do ácido esteárico, os quais são encontrados mais no óleo de palma, e na manteiga e na banha, respectivamente. ^26^

Em nosso estudo, contudo, o ácido palmítico não apresentou correlação significativa com colesterol sanguíneo. Esse foi um resultado inesperado, uma vez que é sabido que o ácido palmítico aumenta os níveis de colesterol total e de LDL. ^27^ Embora os AGSs de hemácias reflitam a ingestão de AGS, ^28^ sabe-se também que carboidratos dietéticos modulam os níveis de AGSs circulantes por meio da lipogênese *de novo* , levando a uma discrepância entre o estado nutricional e a ingestão de AGSs. ^12^ A lipogênese *de novo* encontra-se elevada na obesidade, ^12^ o que poderia explicar a falta de associação entre ácidos graxos dietéticos e das hemácias, observada no presente estudo (Tabela S3). Ainda, a ingestão de ácidos graxos totais e AGSs não foi elevada (29,7 % e 10,2 % da ingestão de energia, respectivamente) e, portanto, a possibilidade de que o ácido palmítico cause hipercolesterolemia nesses níveis de ingestão é baixa. ^20^

Por outro lado, o ácido esteárico mostrou uma correlação inversa significativa com o colesterol total, colesterol não-HDL, apoB, e triglicerídeos. Um ECR relatou que os efeitos dos ácidos esteárico, oleico, e linoleico não apresentaram diferença, sugerindo que o ácido esteárico, diferentemente do ácido palmítico, não aumenta os níveis de LDL. ^29^ Uma metanálise recente mostrou que a substituição do ácido palmítico com ácido esteárico teve pouco ou nenhum efeito sobre o colesterol total, LDL, e apo B. ^27^ Ainda, a substituição dietética do ácido esteárico com AGMI ou AGPI não afetou sobre as concentrações de LDL, HDL, colesterol total ou triglicerídeos, diferentemente da substituição do ácido palmítico com AGMI ou AMPI. Tal fato corrobora a hipótese de que o ácido esteárico dietético pode ser menos deletério que o ácido palmítico quanto aos efeitos sobre o perfil lipídico. Assim, nossos resultados corroboram essa hipótese, uma vez que o ácido esteárico das hemácias mostrou melhor correlação com os lipídios sanguíneos em comparação ao ácido palmítico.

A associação entre AGS circulante e risco cardiovascular tem sido controversa. A metanálise de Chowdhury et al. ^23^ não encontraram relação entre biomarcadores do status de AGS e desfechos coronarianos. Contudo, esses resultados foram criticados, ^30^ uma vez que a meta-análise incluiu estudos que utilizaram diferentes frações lipídicas, que refletem a ingestão de ácidos graxos de diferentes maneiras (por exemplo, os níveis de ácidos graxos plasmáticos de jejum refletem o consumo dos últimos 3-4 dias, enquanto os níveis de ácidos graxos derivados de fosfolipídios refletem a ingestão nos últimos meses). ^23 , 30^ Dos oito estudos incluídos na meta-análise, ^23^ quatro estudos que analisaram fosfolipídios plasmáticos mostraram associações significativas com DAC e mortalidade, ^31 - 34^ sendo as associações mais fortes com ácido palmítico e esteárico.

Além da modulação do metabolismo das lipoproteínas, os AGSs podem influenciar o risco cardiovascular por meio da inflamação. ^12^ Os AGSs atuam como agonistas não microbianos do receptor do tipo Toll 4 (TLR4), ativando vias inflamatórias por meio do o fator de transcrição nuclear kappa B (NF-kB), que exerce um papel crucial na indução de mediadores inflamatórios tais como IL-1β, IL-6, MCP1, TNF-α, entre outros. ^35^ Os AGSs também induzem indiretamente a ativação de TLR4 por meio da produção elevada ade lipopolissacarídeos e toxinas urêmicas pela microbiota intestinal após a ingestão de uma dieta hiperlipídica. Essa endotoxemia metabólica leva a um estresse oxidativo, e à produção de lipídios aterogênicos, LDL oxidado (LDLox) e fosfolipídios oxidados – que levam à resposta inflamatória do complexo CD36-TLR4-TLR6. ^35 , 36^ Ainda, o consumo elevado de AGSs aumenta os níveis de lipídios, de LDL minimamente modificada e de LDLox, que ativam a via inflamatória CD14-TLR4-MD2. ^35^ O consumo elevado de AGSs também pode alterar a composição lipídica do HDL e a capacidade de efluxo do colesterol, diminuindo sua função e aumentando o risco cardiovascular. ^37 , 38^

Nosso estudo corrobora esse fato, ao mostrar que os AGSs das hemácias associam-se independentemente com marcadores inflamatórios. No entanto, essa associação restringiu-se ao ácido esteárico. Sabe-se que o ácido esteárico induz a resposta inflamatória. ^35^ Em uma análise de uma subamostra do PREDIMED, os AGSs plasmáticos e, especificamente, o ácido palmítico, mostraram uma associação positiva com níveis mais altos de moléculas pó-inflamatórias circulantes, particularmente IL-6. ^39^ Por outro lado, Voon et al. ^40^ mostraram que uma dieta rica em ácido palmítico não modificou os níveis de biomarcadores. Contudo, biomarcadores do status de AGSs não foram medidos no estudo. Outros estudos mostraram que AGSs totais estão associados a níveis mais elevados de marcadores inflamatórios, tais como PCR-us e IL-6, ^33 , 34 , 41 , 42^ e risco cardiovascular mais alto. ^31 - 34^ Porém, quando analisados separadamente, as associações tende a ser mais significativas e mais fortes com o ácido esteárico, sugerindo que o ácido palmítico isoladamente não altera o risco cardiovascular na mesma magnitude que o ácido esteárico. ^31 - 34 , 42^

Devido ao seu efeito neutro sobre o metabolismo de lipoproteínas, o ácido esteárico tem sido considerado um substituto dietético para os ácidos graxos *trans* visando redução no risco cardiovascular. ^43^ Contudo, o risco cardiovascular não se resume aos fatores de risco tradicionais principais, ^13^ e vários biomarcadores cardiovasculares potenciais que refletem diferentes aspectos da saúde cardiovascular tem sido estudados. ^3 , 44^ A associação independente do ácido esteárico das hemácias com a PCR-us, IL-6 e TNF-α encontrada em nosso estudo sugere uma ação pró-inflamatória dos ácidos graxos, que leva à disfunção endotelial e ao aumento do risco cardiovascular, independentemente dos efeitos sobre o metabolismo das lipoproteínas, corroborando estudos anteriores. ^31 - 34 , 42^ Contudo, a maioria dos estudos são observacionais. Um ECR recente comparando uma dieta rica em ácido palmítico e uma dieta rica em ácido esteárico mostrou que, apesar dos melhores efeitos sobre o metabolismo lipídico, o ácido esteárico aumentou os níveis de marcadores inflamatórios de baixo grau circulantes. ^45^ Estudos laboratoriais mostram que o ácido esteárico tem efeitos comparáveis aos do ácido palmítico em ativar a cascata de resposta inflamatória TLR4/Nf-κB. ^46^ Um estudo prévio mostrou que o ácido esteárico é um fator contribuinte importante para a lipotoxicidade nas células beta de camundongos, mostrando efeitos mais deletérios que o ácido palmítico na sobrevida de células beta e controle glicometabólico. ^47^ Entretanto, não observamos nenhuma associação entre os AGSs e marcadores glicometabólicos no estudo. Também foi demonstrado que o tratamento de macrófagos polarizados M1 com ácido esteárico aumenta sua susceptibilidade à inflamação e estresse de retículo endoplasmático por meio da inflamação independente de TLR4/2. ^48^ Ainda, o ácido esteárico, em concentrações fisiológicas, mas não o ácido palmítico, induz efeitos lipotóxicos sobre células angiogênicas circulantes, reduzindo, assim sua capacidade de reparo endotelial. ^49^ Além disso, a expressão de genes pró-inflamatórios, induzida por ácido esteárico, aumenta o estresse de retículo endoplasmático e apoptose de células angiogênicas, o que poderia estar associado com dano e disfunção vascular aumentados. ^49^ A associação do ácido esteárico encontrada no presente estudo também pode ser explicada por seu efeito de aumentar a atividade de estearoil-CoA 1 (SDC1), uma enzima lipogênica associada à disfunção metabólica e inflamação crônica de baixo grau, embora os mecanismos pelos quais a SCD1 aumenta a inflamação sejam desconhecidas. ^50^ Contudo, não avaliamos polimorfismos de nucleotídeo único ou atividade da SCD1 em nosso estudo.

Em conjunto, os resultados de nosso estudo e da literatura corroboram o efeito pró-inflamatório do ácido esteárico. Sabe-se que a PCR-us, o IL-6, e o TNF-α não são apenas marcadores do sistema de inflamação, como também biomarcadores da disfunção endotelial. ^51 , 52^ A disfunção endotelial é o principal marco de DCV e está associada com pior prognóstico independentemente dos fatores de risco. ^1^ De fato, níveis elevados de ácido esteárico podem aumentar o risco inflamatório cardiovascular, independentemente dos níveis de colesterol sanguíneo. Nosso estudo tem limitações devido à sua natureza transversal, o tamanho amostral, e a falta de desfechos clínicos. Os pontos fortes do estudo incluem o uso de ácidos graxos das hemácias como um biomarcador do status de AGS, e o uso de biomarcadores de inflamação e de disfunção endotelial. A razão pelo qual os ácidos graxos de hemácias não se correlacionaram com ácidos graxos da dieta pode ser atribuída a vários vieses inerentes aos inquéritos alimentares, sugerindo a vantagem de se utilizar biomarcadores do status de ácidos graxos, particularmente ácidos graxos das hemácias, que refletem a ingestão de ácidos graxos nos últimos três meses aproximadamente.

## Conclusões

Nossos resultados mostraram que o ácido esteárico de hemácias associa-se independentemente com biomarcadores de inflamação e de disfunção endotelial em indivíduos com um ou mais fator de risco cardiovascular.
