# Supplementary material for: Ácido Esteárico (mas não o Ácido Palmítico) está Associado a Biomarcadores Inflamatórios e de Disfunção Endotelial em Indivíduos em Risco Cardiovascular
Source: Arq Bras Cardiol. 2023 Aug 10;120(8):e20220598. [Article in Portuguese] doi: 10.36660/abc.20220598 (PMC10519248; doi:10.36660/abc.20220598)
Supplement: Translation

# Translation: Stearic Acid, but not Palmitic Acid, is Associated with Inflammatory and Endothelial Dysfunction Biomarkers in Individuals at Cardiovascular Risk

**Authors:** Gustavo Henrique Ferreira Gonçalinho, Geni Rodrigues Sampaio, Rosana Aparecida Manólio Soares-Freitas, Nágila Raquel Teixeira Damasceno

**Affiliations:** 1 Brasil Universidade de São Paulo – Departamento de Nutrição, São Paulo, SP – Brasil

**Keywords:** Fatty Acids, Endothelium, Inflammation, Biomarkers, Cardiovascular Diseases

## Abstract

**Background:**

Several studies have associated dietary saturated fatty acids (SFAs) with cardiovascular risk but there are still many controversies. Most of these studies have focused on the effects of palmitic acid on circulating lipids. Stearic acid usually shows a neutral effect on blood lipids, however, there is a lack of clinical studies assessing the link with inflammatory and endothelial dysfunction markers.

**Objective:**

To evaluate the association of red blood cell (RBC) SFA (palmitic and stearic acids) with circulating inflammatory and endothelial dysfunction biomarkers.

**Methods:**

Cross-sectional study of 79 adults of both sexes with at least one cardiovascular risk factor but without previous events (acute myocardial infarction or stroke). Plasma biomarkers – lipids, glucometabolic markers, high-sensitivity C-reactive protein (hs-CRP), interleukin-6 (IL-6), interleukin-10 (IL-10), monocyte chemoattractant protein-1 (MCP-1), and tumor necrosis factor-α (TNF-α) – and RBC palmitic and stearic fatty acids were analyzed. The associations were assessed by correlation and multiple linear regression analyses, with statistical significance set at p < 0.05.

**Results:**

Palmitic acid showed no significant associations with traditional cardiovascular risk factors or inflammatory markers. Stearic acid, on the other hand, was inversely correlated with blood cholesterol and triglycerides, but independently associated with hs-CRP, IL-6, and TNF-α.

**Conclusion:**

Stearic acid is associated with inflammatory and endothelial dysfunction biomarkers in individuals with at least one cardiovascular risk factor.

## Introduction

The role of inflammation in cardiovascular diseases (CVDs) has gained much emphasis in the literature. Patients who are on intensive cholesterol-lowering therapy using statins, ezetimibe, and PCSK9 inhibitors may have a so-called “residual inflammatory risk”, in which patients with higher levels of inflammatory markers such as tumor necrosis factor-α (TNF-α), interleukin-6 (IL-6) and C-reactive protein (CRP), despite cholesterol lowering, may experience more cardiovascular events when compared to patients with lower levels.^1^Several biomarkers of inflammation have been associated with the incidence, prevalence, severity, and prognosis of CVDs. They may reflect CVD from a different perspective than that of traditional risk factors since many studies have shown independence in the association of these markers.^2^Also, the increase in these circulating inflammatory markers has been associated with increased levels of vascular cell adhesion molecule-1 (VCAM-1), intercellular adhesion molecule-1 (ICAM-1), and E-selectin, indicators of impaired endothelial function. This may explain the relationship between inflammation and CVD.^3 , 4^Therefore, inflammatory markers such as CRP and IL-6 may also indicate endothelial dysfunction.^2 - 4^

Current evidence suggests that high intake of dietary saturated fatty acids (SFAs) is associated with increased cardiovascular risk,^5^and limiting SFA consumption to reduce this risk is still recommended by the recently published nutrition guidelines of the American Heart Association^6^and the Brazilian Society of Cardiology.^7^Mechanisms of how SFAs contribute to atherosclerosis progression and increase cardiovascular risk include mainly increased blood total cholesterol, low-density lipoprotein-cholesterol (LDL-c), triglycerides,^8^and inflammation.^9 - 11^

Among the mechanisms demonstrated in the literature, it has been shown that SFAs are activators of Toll-like receptors (TLRs) in macrophages, triggering the inflammatory signaling pathway and subsequently, immunometabolic dysfunctions found in cardiometabolic diseases.^12^Clinical evidence of how SFAs influence inflammation and consequently CVD risk remains controversial due to different methods.^12^Most studies have investigated the effects of palmitic acid; studies focusing on stearic acid have shown a nearly neutral effect on human health from the lipoprotein point of view. Studies on the influence of this SFA on biomarkers of endothelial function and inflammation are lacking.^12^Food surveys, commonly used in studies that assessed the effects of SFAs on health, contain several biases, and red blood cells (RBC) fatty acids have been used as biomarkers of the nutritional status of these nutrients,^13^and may provide more consistent evidence of how SFAs are associated with inflammation and endothelial function in humans.

Therefore, the objective of the present study is to evaluate the association of RBC SFAs (palmitic and stearic acids) with circulating biomarkers of inflammation and endothelial dysfunction in individuals with cardiovascular risk factors but without established CVD.

## Methods

### Study design and participants

This was a cross-sectional study of baseline data from the CARDIONUTRI clinical trial (ReBEC: RBR-2vfhfv). The participants were recruited from the outpatient clinic at the University of São Paulo Hospital. Inclusion criteria were individuals of both sexes, 30 to 74 years, with at least one cardiovascular risk factor, and no previous cardiovascular events (acute myocardial infarction or stroke). Exclusion criteria were individuals with acute or severe chronic diseases, infectious diseases, pregnant, and/or lactating women. Screened individuals were submitted to a short phone interview to assess inclusion and exclusion criteria. Additionally, individuals underwent an electrocardiogram conducted by a cardiologist and those with alterations suggesting previous cardiovascular events were excluded. Three hundred and seventy-four individuals were recruited for the study from 2011 to 2012. Two individuals declined after being informed about the study design. Fourteen were excluded due to altered electrocardiograms and two due to recent HIV diagnosis. At the end of the recruitment, 356 individuals were included in the CARDIONUTRI trial. For the present study analysis, only participants who had laboratory information on inflammatory markers (plasma cytokines) were included, resulting in 79 subjects ( Figure 1 ).

**Figure 1:** – Study flow chart.

### Clinical and nutritional assessment

Medical history of non-communicable chronic diseases and current medication use was self-reported. Physical examination included body mass index (BMI), waist circumference, and blood pressure assessment. Dietary intake was obtained through three 24-hour recalls and assessed in the Food Processor software (ESHA Research, 2012), with subsequent energy adjustment.^14^Cardiovascular risk was assessed by the Framingham Risk Score,^15 , 16^and individuals were classified as at low, moderate, or high risk. Diabetes was considered a coronary artery disease (CAD) equivalent.^17^

### Biochemical analysis

Blood was collected after a 12-h fast into EDTA tubes (1.0 mg/mL), and erythrocytes were separated from plasma by centrifugation; both were frozen at -80 °C immediately after collection. Protease inhibitors (10 µg/mL of aprotinin, 10 µg/mL of benzamidine, and 5 µg/mL of phenylmethylsulfonyl fluoride) and butylated hydroxytoluene (BHT, 100 µg/mL) were added to preserve samples. All samples were divided into aliquots to avoid repeated defrost cycles and stored at -80 °C until analyses. Plasma total cholesterol, high-density lipoprotein-cholesterol (HDL-c), triglycerides, glucose (Labtest Diagnostica SA, MG, Brazil), non-esterified fatty acids (NEFA), apolipoproteins (Apo) A-I and B (Wako Chemicals USA Inc., Richmond, VA, USA), and high sensitivity C-reactive protein (hs-CRP) (Diagnostic System Laboratories, Inc., Webster, TX, USA) were measured by commercial kits. Plasma interleukin (IL)-6, IL-10, monocyte chemoattractant protein-1 (MCP-1), and TNF-α were measured using a Bio-PlexTM Human Cytokine 17-plex Assay ELISA kit (Bio-Rad Laboratories, CA, USA). Serum insulin was measured by a Human Insulin ELISA kit (Life Technologies, NY, USA). LDL-c was calculated using the Friedewald equation.^18^Insulin sensitivity was measured by the Homeostasis Model Assessment – Insulin Resistance (HOMA-IR), and insulin resistance was diagnosed if the individuals presented any of the following conditions: BMI > 28.9 kg/m^2^, HOMA-IR >4.65, or BMI >27.5 kg/m^2^and HOMA-IR >3.60.^19^

### Red blood cell fatty acids analysis

Analysis of RBC fatty acids was performed by gas chromatography as described elsewhere.^13^After plasma separation, 300 μL of RBC were washed with 5 mL of phosphate-buffered saline (PBS) solution (pH 7.4) four times. The precipitate was transferred to screw-cap tubes, to which 1.75 mL of methanol, 50 µL of an internal standard solution containing 1 mg tridecanoic acid (C13:0)/1 mL hexane, and 100 µL of acetyl chloride were added. Thereafter, the solution was vortexed and heated in a water bath at 90 °C for one hour. After that, 1.5 mL of hexane was added, and the solution was homogenized for 1 min. The samples were centrifuged at 1500× g, 4 °C for two minutes, and 800 µL of the supernatant was transferred to a different tube. This step was repeated with the addition of 750 µL of hexane. The tubes containing the collected supernatants were placed in a centrifugal concentrator at 40 °C for 20 minutes. Then the FA methyl esters were dissolved in 150 µL of hexane and transferred to a glass insert in a vial. In total, 19 RBC fatty acids were detailed, which included SFA, monounsaturated FA (MUFA), and polyunsaturated fatty acids (PUFA). Among the RBC fatty acids we analyzed palmitic, stearic, arachidic, behenic, lignoceric, palmitoleic, oleic, gondoic, erucic, nervonic, linoleic, γ-linoleic, eicosadienoic, dihomo-γ-linoleic, arachidonic, 13,16-docosadienoic, α-linoleic, eicosapentaenoic, and docosahexaenoic acids. In the present study, we focused our analyses on SFAs, more specifically on stearic and palmitic acids, which were the most predominant SFA in erythrocytes.

### Statistical analysis

The distribution of variables was assessed through the Kolmogorov-Smirnov test. Sample characteristics are presented as mean and standard deviation (SD) or median and interquartile range (IQR) for quantitative variables, depending on the variables’ distribution, and frequency (n) and percentage (%) for categorical variables.

The relationship of SFAs with cardiometabolic parameters and cytokines was assessed through Pearson’s and Spearman’s correlations depending on the variables’ distribution.

To assess the influence of palmitic and stearic acids on inflammation biomarkers, multiple linear regressions were applied using the inflammation biomarkers (hs-CRP, IL-6, IL-10, MCP-1, TNF-α) as dependent variables, and palmitic or stearic acid as the independent variable in addition to the adjustment variables (age, sex, smoking, total cholesterol, systolic blood pressure, glucose, and BMI). All regression assumptions were fulfilled ( *i.e* ., no multicollinearity, homoscedasticity, normally distributed and independent errors, independence of the outcome variables, and linearity of the variables).

All statistical tests were two-sided, with p < 0.05 considered statistically significant, and performed on the SPSS software version 20.0.

## Results

Characteristics of the participants are summarized in Table 1 and Table S1. The sample was composed mainly of women (59.5%) and had a mean age of 51.0 ± 10.3 years. The prevalence of self-reported chronic disease in the sample was 60.8% of individuals with hypertension, 52.2% of dyslipidemia, and 25.3% of type 2 diabetes mellitus.

**Table 1:** – Clinical and biochemical characteristics of the participants

Variables	Mean/Median	SD/IQR
Age, *years*	51.0	10.3
Body mass index, *kg/m* ^*2*^	31.5	6.1
Waist circumference, *cm*	101.5	13.1
Systolic blood pressure, *mmHg*	135.2	19.2
Diastolic blood pressure, *mmHg*	81.8	9.9
Heart rate, *bpm*	67.8	13.0
Total cholesterol, *mg/dL*	199.8	43.9
HDL-c, *mg/dL*	35.8	10.2
LDL-c, *mg/dL*	134.8	41.4
Total cholesterol:HDL-c	6.0	4.0 – 7.0
Non-HDL-c, *mg/dL*	164.4	45.0
ApoA-I, *mg/dL*	126.9	25.7
ApoB, *mg/dL*	101.8	26.9
Triglycerides, *mg/dL*	129.0	94.0 – 188.0
Non-esterified fatty acids, *mEq/dL*	0.62	0.25
Glucose, *mg/dL*	96.0	89.0 – 108.0
Insulin, *μUI/mL*	17.7	8.4
HOMA-IR	4.4	2.4
hs-CRP, *mg/L*	2.8	1.2 – 5.6
IL-6, *pg/mL*	5.3	3.3 – 9.0
IL-10, *pg/mL*	4.0	2.8 – 10.5
MCP-1, *pg/mL*	15.1	10.5 – 21.6
TNF-α, *pg/mL*	15.2	8.2 – 25.9

HDL: high-density lipoprotein, LDL: low-density lipoprotein, Apo: apolipoprotein, HOMA-IR: Homeostasis Model Assessment – Insulin Resistance, IL: interleukin, hs-CRP: high-sensitivity C-reactive protein; TNF-α: tumor necrosis factor-α, MCP-1: monocyte chemoattractant protein-1. SD: standard deviation; IQR: interquartile range

Regarding the Framingham Risk Score, most of the participants were classified as high cardiovascular risk (51.9%), followed by moderate risk (34.2%) and low risk (13.9%). The sample presented several cardiovascular risk factors, such as elevated BMI and waist circumference, hypercholesterolemia, low HDL-c, hyperglycemia, hyperinsulinemia, HOMA-IR, and elevated hs-CRP ( Table 1 ).

The RBC fatty acids profile is described in Table 2 . The most abundant RBC fatty acids were palmitic and stearic acids. The most abundant MUFA was oleic acid (C18:1 n-9). Among PUFA, the most abundant FA were linoleic acid and arachidonic acid of the n-6 family and docosahexaenoic acid (DHA) of the n-3 family.

**Table 2:** – Profile of red blood cell fatty acids of the participants

Variables	Mean/Median	SD/IQR
**SFA, *%***		
C16:0 (Palmitic acid)	44.02	4.87
C18:0 (Stearic acid)	26.12	4.45
C20:0 (Arachidic acid)	0.73	0.18
C22:0 (Behenic acid)	1.29	0.51
C24:0 (Lignoceric acid)	0.27	0.14 – 0.77
**MUFA, *%***		
C16:1 n-7 (Palmitoleic acid)	0.33	0.22 – 0.76
C18:1 n-9 (Oleic acid)	8.99	3.71
C20:1 n-9 (Gondoic acid)	0.08	0.05 – 0.14
C22:1 n-9 (Erucic acid)	0.14	0.10 – 0.21
C24:1 n-9 (Nervonic acid)	1.36	0.67
**PUFA n-6, *%***		
C18:2 n-6 (Linoleic acid)	4.34	1.91
C18:3 n-6 (γ-linoleic acid)	0.17	0.12 – 0.24
C20:2 n-6 (Eicosadienoic acid)	0.10	0.07 – 0.17
C20:3 n-6 (Dihomo- γ-linoleic acid)	0.54	0.36
C20:4 n-6 (Arachidonic acid)	3.43	2.60
C22:2 n-6 (13,16-Docosadienoic acid)	0.39	0.21
Total n-6	9.18	4.07
**PUFA n-3, *%***		
C18:3 n-3 (α-linolenic acid)	1.64	1.01 – 2.28
C20:5 n-3 (Eicosapentaenoic acid)	0.24	0.14
C22:6 n-3 (Docosahexaenoic acid)	3.91	1.31
Total n-3	6.16	2.03

MUFA: monounsaturated fatty acids, PUFA: polyunsaturated fatty acids, SD: standard deviation; IQR: interquartile range.

The nutritional composition of the participants’ diet and its correlation with inflammatory markers are described in Table S2 and Table S3. The mean consumption of SFAs was 10.2 ± 3.9 %, with palmitic and stearic acids consumption of 6.4 ± 4.0 and 3.0 ± 2.4 g, respectively. There was no statistically significant correlation between dietary SFA and inflammatory markers.

The correlations between RBC SFA (palmitic and stearic acids) are described in Table 3 . Total cholesterol, non-HDL-c, total cholesterol: HDL-c ratio, apoB, and triglycerides were inversely correlated with stearic acid. However, hs-CRP, IL-6, IL-10, MCP-1, and TNF-α were positively correlated with stearic acid.

**Table 3:** – Correlations between red blood cell fatty acids and cardiovascular biomarkers

Variables	Palmitic acid		Stearic acid	
	
	r	p-value	r	p-value
Sex	0.079	0.490	0.197	0.082
Age, *years*	0.062	0.585	0.163	0.151
Body mass index, *kg/m* ^*2*^	0.013	0.907	-0.036	0.756
Waist circumference, *cm*	0.021	0.857	-0.076	0.506
Systolic blood pressure, *mmHg*	0.034	0.767	-0.113	0.323
Diastolic blood pressure, *mmHg*	0.024	0.833	-0.151	0.184
Heart rate, *bpm*	0.049	0.667	0.072	0.531
Total cholesterol, *mg/dL*	-0.032	0.779	-0.253	0.024
HDL-c, *mg/dL*	0.089	0.434	0.198	0.080
LDL-c, *mg/dL*	-0.088	0.453	-0.211	0.069
Total cholesterol:HDL-c*	-0.083	0.468	-0.330	0.003
Non-HDL-c, *mg/dL*	-0.052	0.649	-0.292	0.009
ApoA-I, *mg/dL*	0.155	0.173	-0.023	0.843
ApoB, *mg/dL*	0.009	0.936	-0.290	0.009
Triglycerides, mg/dL*	-0.011	0.927	-0.244	0.030
Non-esterified fatty acids, *mEq/dL*	0.283	0.016	-0.007	0.954
Glucose, mg/dL*	-0.114	0.317	-0.034	0.768
Insulin, *μUI/mL*	0.133	0.298	-0.220	0.083
HOMA-IR	0.093	0.467	-0.210	0.099
hs-CRP, mg/L*	0.114	0.325	0.286	0.012
IL-6, pg/mL*	0.017	0.881	0.340	0.002
IL-10, pg/mL*	0.029	0.833	0.357	0.007
MCP-1, pg/mL*	0.034	0.769	0.245	0.030
TNF-α, pg/mL*	0.009	0.938	0.364	0.002

HDL: high-density lipoprotein, LDL: low-density lipoprotein, Apo: apolipoprotein, HOMA-IR: Homeostasis Model Assessment – Insulin Resistance, IL: interleukin, hs-CRP: high-sensitivity C-reactive protein; TNF-α: tumor necrosis factor-α, MCP-1: monocyte chemoattractant protein-1, SD: standard deviation; IQR: interquartile range; the correlation analysis was performed using Pearson’s test for parametric variables and Spearman’s test (*) for non-parametric variables

In the multiple linear regression models ( Table 4 ), palmitic acid did not present any statistically significant association with inflammatory biomarkers, whereas stearic acid was independently factor associated with hs-CRP, IL-6, and TNF-α when adjusted by age, sex, smoking status, total cholesterol, systolic blood pressure, plasma glucose, and BMI.

**Table 4:** – Associations between red blood cell fatty acids and circulating inflammation and endothelial dysfunction biomarkers

Dependent variables	R^**2**^	β	SE	95% CI for β		p-value

				Lower	Upper	
**Palmitic acid (C16:0)**						
hs-CRP	0.079	0.165	0.162	-0.158	0.489	0.311
IL-6	0.110	0.132	1.789	-3.438	3.702	0.941
IL-10	0.127	0.728	2.134	-3.589	5.024	0.735
MCP-1	0.091	0.399	2.184	-3.957	4.756	0.855
TNF-α	0.127	0.112	1.279	-2.445	2.669	0.930
**Stearic acid (C18:0)**						
hs-CRP	0.162	0.545	0.194	0.157	0.933	**0.007**
IL-6	0.173	4.620	2.026	0.577	8.664	**0.026**
IL-10	0.186	4.370	2.359	-0.379	9.120	0.070
MCP-1	0.116	3.584	2.526	-1.454	8.623	0.160
TNF-α	0.194	3.230	1.443	0.345	6.116	0.029

Multiple linear regression models adjusted by age, sex, smoking status, total cholesterol, systolic blood pressure, glucose, and body mass index. The models were composed by one of the dependent variables – high-sensitivity C-reactive protein (hs-CRP); interleukin-6 (IL-6), IL: interleukin-10 (IL-10), monocyte chemoattractant protein-1 (MCP-1) and tumor necrosis factor-α (TNF-α) and independent variables (palmitic acid or stearic acid and the adjustment variables), 95%CI: 95% confidence interval

## Discussion

The main finding of our study ( Central Illustration ) was that stearic acid was independently associated with circulating biomarkers of endothelial dysfunction and inflammation despite inverse correlations with atherogenic blood lipids in individuals with cardiovascular risk factors without CVD. Additionally, there was statistically significant association of palmitic acid with any of the biomarkers assessed.

**Central Illustration:** : Stearic Acid, but not Palmitic Acid, is Associated with Inflammatory and Endothelial Dysfunction Biomarkers in Individuals at Cardiovascular Risk

Hs-CRP: high-sensitivity C-reactive protein; IL: Interleukin; CVD: cardiovascular disease.

Limiting SFA intake for cardiovascular risk lowering is recommended by recently published nutrition guidelines.^6 , 7^A meta-analysis of randomized controlled trials (RCT) showed that palm oil rich in palmitic acid raised LDL-c by 9 mg/dL when compared to low-SFA vegetable oils.^20^This can be translated into a 6% higher risk of coronary artery disease (CAD).^21^Another meta-analysis showed that dietary SFA reduction decreased LDL-c by 5 mg/dL in children and adolescents.^22^Importantly, these effects occur when fatty acid intake range is within 20 to 30% of total daily energy intake. When the fatty acid energy intake is greater than 30%, the magnitude of the LDL-c raising effect by SFA increased to 24 mg/dL.^20^However, other studies showed weak associations between SFA intake and CVD outcomes.^23 , 24^These controversies could be explained by the replacement of SFAs with refined carbohydrates,^25^and comparing the effects of SFA with *trans-* fatty acid, animal fat, or coconut oil,^20^could make it difficult to evaluate the real effect of SFA. Furthermore, most observational studies do not distinguish the overall effects of palmitic acid and stearic acid, which are found more in palm oil and butter and lard, respectively.^26^

In our study, however, palmitic acid did not have any significant correlation with blood cholesterol. It was an unexpected result since it is known that palmitic acid raises total cholesterol and LDL-c.^27^Although red blood cells’ SFAs reflect the SFA intake,^28^it is also known that carbohydrate intake modulates circulating SFA levels through *de novo* lipogenesis, causing a discrepancy between nutritional status and SFA intake.^12^ *De novo* lipogenesis is found elevated in obesity,^12^which could explain the lack of association between RBC and dietary fatty acid found in the present study (Table S3). Furthermore, total fatty acids and SFA intake was not high (29.7% energy and 10.2% energy, respectively), making it unlikely that palmitic acid causes hypercholesterolemia at these intake levels.^20^

Stearic acid, on the other hand, showed a significant inverse correlation with total cholesterol, non-HDL-c, apoB, and triglycerides. One RCT reported that the effects of stearic, oleic, and linoleic acids on lipoproteins had no difference, suggesting that stearic acid, unlike the palmitic acid, does not raise LDL-c.^29^A recent meta-analysis showed that replacing dietary palmitic acid with stearic acid had little or no effect on total cholesterol, LDL-c, and apoB.^27^Furthermore, dietary substitution of stearic acid with MUFA or PUFA showed that concentrations of LDL-c, HDL-c, total cholesterol, or triglycerides were not affected, contrasting with the effects when palmitic acid was replaced by MUFA or PUFA. This corroborates the hypothesis that dietary stearic acid might be less detrimental than palmitic acid on blood lipids. Thus, our results corroborate this hypothesis, since RBC stearic acid showed more favorable correlations with blood lipids compared to palmitic acid.

The association between circulating SFA and cardiovascular risk has been controversial. The meta-analysis of Chowdhury et al.^23^found no relationship between SFA status biomarkers and coronary outcomes. However, these results have been criticized,^30^since the meta-analysis included studies that used different lipid fractions, which reflect dietary fatty acid intake variously (e.g., fasting plasma fatty acid reflects intake over the last 3-4 days, whilst plasma phospholipid fatty acids reflect intake over the last months).^23 , 30^Of the eight studies included in the meta-analysis,^23^four studies analyzing plasma phospholipids, showed significant associations with coronary heart disease and mortality,^31 - 34^with stronger associations with palmitic and stearic acids.

In addition to modulation of lipoprotein metabolism, SFAs may influence cardiovascular risk through inflammation.^12^SFAs act as non-microbial Toll-like receptor 4 (TLR4) agonists, triggering inflammatory pathways through nuclear factor kappa B (NF-κB), which plays a crucial role in the induction of inflammatory mediators such as IL-1β, IL-6, MCP1, TNF-α, and others.^35^SFA also trigger TLR4 activation indirectly by the overproduction of lipopolysaccharides and uremic toxins by gut microbiota after a high-fat dietary intake. This metabolic endotoxemia leads to oxidative stress thereby producing atherogenic lipids – oxidized LDL (oxLDL) and oxidized phospholipids – which trigger the CD36-TLR4-TLR6 complex inflammatory response.^35 , 36^Furthermore, high SFA consumption increases lipemia and minimally modified LDL and oxLDL levels, which activate CD14-TLR4-MD2 inflammatory pathway.^35^In addition, high SFA consumption may alter HDL lipid composition and cholesterol efflux capacity, decreasing its function and increasing cardiovascular risk.^37 , 38^

In line with that, our study showed that RBCSFA are independently associated with inflammatory markers. However, this association was restricted to stearic acid. Palmitic acid is known to induce the inflammatory response.^35^In an analysis of a PREDIMED sub-sample, plasma SFA and specifically palmitic acid were positively associated with higher levels of circulating pro-inflammatory molecules, particularly IL-6.^39^On the other hand, Voon et al.^40^showed that a high palmitic acid diet did not modify inflammatory biomarkers. However, SFA status biomarkers were not measured in the study. Other studies showed that circulating total SFAs are associated with higher inflammatory markers circulating levels such as hs-CRP and IL-6,^33 , 34 , 41 , 42^and higher cardiovascular risk^31^but when analyzed separately, the associations tend to be more significant and stronger with stearic acid, suggesting that palmitic acid alone does not modify cardiovascular risk in the same magnitude as with stearic acid.^31 - 34 , 42^

Due to its neutral effect on lipoprotein metabolism, stearic acid has been considered a dietary substitute for *trans* fatty acids for cardiovascular risk reduction.^43^However, cardiovascular risk goes beyond major traditional risk factors,^13^and several emerging potential cardiovascular biomarkers that reflect different aspects of cardiovascular health are being studied.^3 , 44^The independent association of RBC stearic acid with hs-CRP, IL-6, and TNF-α found in our study suggests a pro-inflammatory and endothelial dysfunction-causing action, and consequently, a cardiovascular risk-raising effect of the fatty acids, independently of their effects on lipoprotein metabolism, corroborating the aforementioned studies.^31 - 34 , 42^Nonetheless, most of the studies are observational. A recent RCT comparing a palmitic acid-rich diet and a stearic acid-rich diet showed that although better lipid metabolism effects, stearic acid increased the circulating levels of low-grade inflammation markers.^45^Laboratory studies show that stearic acid has comparable effects to palmitic acid in activating the TLR4/Nf-κB inflammatory response cascade.^46^A previous study demonstrated that stearic acid is a major contributor to lipotoxicity in beta cells of mice, showing more detrimental effects than palmitic acid in beta cell survival and glucometabolic control.^47^However, we did not observe any association between SFA and glucometabolic markers in the study. It was also shown that treatment of M1polarized macrophages with stearic acid increases their susceptibility to inflammation and endoplasmic reticulum (ER) stress through TLR4/2-independent inflammation.^48^Additionally, stearic acid at physiological concentrations, but not palmitic acid, induces lipotoxic effects on circulating angiogenic cells (CACs), thereby reducing its endothelial repair capacity.^49^Moreover, the expression of pro-inflammatory genes induced by stearic acid increases ER stress and CAC apoptosis, which could be associated with increased vascular damage and dysfunction.^49^The association of stearic acid found in the present study can also be explained by its effect of increasing the activity of stearoyl-CoA desaturase 1 (SCD1), a lipogenic enzyme associated with metabolic dysfunction and chronic low-grade inflammation, but the mechanisms which SCD1 increases inflammation are unknown.^50^However, we did not evaluate single nucleotide polymorphisms or SCD1 activity in our study.

Together, the findings of our study and the cited literature corroborate the pro-inflammatory effect of stearic acid. It is known that hs-CRP, IL-6, and TNF-α are not only markers of systemic inflammation but also endothelial dysfunction biomarkers.^51 , 52^Endothelial dysfunction is the main hallmark of CVD and is associated with worse prognostic independently of risk factors.^1^Accordingly, high levels of stearic acid might increase residual inflammatory cardiovascular risk independently of blood cholesterol levels. Our work has limitations due to its cross-sectional nature, the small sample, and the lack of clinical outcomes. The strengths of the study include the use of RBC fatty acids a biomarker of SFA status and the use of inflammation and endothelial dysfunction biomarkers. The reason why RBC fatty acids were not correlated with dietary FA may be attributed to various biases embedded in food surveys, suggesting the advantage of using FA status biomarkers, specifically RBC fatty acids, which reflect FA intake approximately over the last three months.

## Conclusions

In conclusion, our findings showed that RBC stearic acid is independently associated with inflammatory and endothelial dysfunction biomarkers in individuals with at least one cardiovascular risk factor.
